# A Scoping Review of the Challenges and Future Perspectives in the Use of Alpha-Emitters for Metastatic Ovarian Cancer

**DOI:** 10.3390/molecules31061019

**Published:** 2026-03-18

**Authors:** Lu Lucy Xu, Satyendra Kumar Singh, Nelli Gaspar, Jinda Fan, Benjamin L. Viglianti, Kurt R. Zinn

**Affiliations:** 1 Department of Biomedical Engineering, Michigan State University, East Lansing, MI 48824, USA; xulu2@msu.edu (L.L.X.); singhs54@msu.edu (S.K.S.); 2 Institute for Quantitative Health Science and Engineering, Michigan State University, East Lansing, MI 48824, USA; nelli.gaspar@icloud.com (N.G.); fanjinda@msu.edu (J.F.); 3 College of Human Medicine, Michigan State University, East Lansing, MI 48824, USA; 4 Department of Pharmacology and Toxicology, Michigan State University, East Lansing, MI 48824, USA; 5 Department of Radiology, Michigan State University, East Lansing, MI 48824, USA; 6 Department of Chemistry, Michigan State University, East Lansing, MI 48824, USA; 7 Department of Radiology, University of Michigan, Ann Arbor, MI 48109, USA; bviglia@med.umich.edu; 8 Department of Small Animal Clinical Sciences, College of Veterinary Medicine, Michigan State University, East Lansing, MI 48824, USA

**Keywords:** targeted alpha-particle therapy, radioimmunotherapy, ovarian cancer, metastasis, clinical trial, pre-clinical, clinical implementation, resistance, dosimetry

## Abstract

Ovarian cancer (OC) is frequently diagnosed at an advanced stage and characterized by high rates of recurrence despite aggressive cytoreductive surgery and chemotherapy. Relapse is driven by microscopic residual tumors that are disseminated most often throughout the peritoneal cavity, posing significant challenges with conventional systemic therapy. Targeted alpha-particle therapy (TAT) combines molecular targeting with alpha-emitting radionuclides to deliver highly potent and localized cellular damage, uniquely suited for the eradication of small OC tumor clusters within the peritoneal cavity. We conducted an extensive literature search for clinical trials (clinicaltrials.gov) and pre-clinical studies (PubMed, Scopus, Google Scholar) between September 2025 and November 2025. Peer-reviewed articles published in English over the past 20 years that used OC mouse models with reported treatment data were included. Review articles without original data and clinical trials that have been terminated or withdrawn were excluded. In this review, we (1) summarize the biological and physical rationale supporting the use of TAT in OC, (2) discuss the relevant molecular and immunological anti-tumor mechanisms, and (3) critically evaluate early treatment outcomes of 19 pre-clinical and four clinical studies with respect to efficacy, safety, and feasibility. Despite the progress and promising survival outcomes, several challenges remain, including heterogeneous antigen expression, delivery and retention within the peritoneal cavity, off-target toxicity, radiation resistance, radionuclide availability, dosimetry uncertainties, and limitations in clinical trial design. We highlight future directions to overcome these barriers and the continued multidisciplinary efforts essential to translate TAT into effective clinical strategies to treat advanced stages of OC and other solid tumors resistant to conventional treatment. This work was supported with funding available to Kurt R. Zinn as the Hickman Family Endowed Chair in Oncology at Michigan State University.

## 1. Introduction

Gynecological malignancies, including cervical, endometrial, and ovarian cancers (OC), represent a significant global health burden, accounting for over 15% of cancer-related morbidity and mortality among women worldwide. Even with the development of HPV vaccines, the incidence is predicted to increase by almost 50% and the number of deaths to increase by 77% by 2050, with OC representing the highest mortality [1]. Despite advances in early detection and multi-modal therapy, OC remains the most lethal and is responsible for more deaths than other gynecological malignancies [2]. Most OC patients are diagnosed at an advanced stage due to the absence of early, non-specific symptoms and lack of reliable screening tools [3]. Approximately 70% of OC patients present with disseminated peritoneal metastases at diagnosis, with a 5-year survival rate of 31% for stage 4 [4,5]. Even after achieving complete clinical remission following cytoreductive surgery and platinum-based systemic chemotherapy or hyperthermic intraperitoneal chemotherapy (HIPEC), recurrence occurs in nearly 70–80% of advanced-stage cases [6]. This underscores the persistent challenge of residual microscopic disease and chemo-resistant OC tumor [7]. Although similar issues of recurrence and metastatic relapse are seen in endometrial and cervical cancers, the occurrence is reduced, and loco-regional control remains the major determinant of long-term survival [8,9]. This highlights the critical need for therapeutic strategies that can effectively eradicate minimal residual disease to improve patient outcomes in metastatic OC.

Radioimmunotherapy (RIT) emerged from the principle of coupling the cytotoxic potential of localized ionizing radiation with the specificity of molecular targeting vectors [10]. Early successes were exemplified by ^90^Y-ibritumomab tiuxetan (Zevalin^®^) and ^131^I-tositumomab (Bexxar^®^) for the treatment of hematological malignancies, demonstrating the therapeutic value of selective radioligand delivery combined with radionuclides [11,12]. The extension of these RIT strategies to solid tumors, including OC, is limited by the heterogeneity and downregulation of specific antigen expression [13,14], poor tumor perfusion [15], and limited penetration of large antibodies into the tumor microenvironment [16,17]. The beta-particle emitters (LET, 0.1–1 keV/μm) used in conventional RIT have relatively long path lengths (up to several mm), which may result in suboptimal dose deposition within smaller tumor clusters and increased toxicity to surrounding healthy tissues. In contrast, alpha-particle emitters deliver ionizing radiation with high linear energy transfer (LET, 50–230 keV/μm) and have a shorter path length (<100 μm) [18]. By comparison, the path length corresponds to only a few cells in diameter, resulting in a higher efficacy [19,20]. This unique profile allows for highly localized and potent cytotoxicity by inducing irreparable dsDNA (double-stranded DNA) breaks, largely independent of oxygenation or cell cycle status [21,22,23]. These properties make alpha-emitters well-suited for targeting disseminated micro-metastases and circulating tumor cells, which are hallmarks of advanced and recurrent OC. Clinical success of targeted alpha-particle therapy (TAT) has been shown for ^223^RaCl_2_ for bone metastases from prostate cancer [24]. Emerging clinical trials of ^225^Ac-, ^213^Bi-, and ^212^Pb-labeled antibodies and peptides have expanded interest in TAT across multiple solid tumor settings, primarily prostate in men [25,26,27]. Comparably, TAT for female gynecological malignancies has been largely understudied compared with the male-predominant malignancies.

This review explores the current landscape, challenges, and future perspectives of alpha-particle-emitting radiopharmaceuticals in gynecological cancers, particularly for metastatic OC. First, we summarize the biological and clinical rationale for alpha-emitters in the context of ovarian cancer pathophysiology. This is followed by an analysis of pre-clinical and clinical studies using various alpha-emitting radionuclides (properties listed in Table 1) and targeting vectors (e.g., antibodies, antibody fragments, peptides, and nanoparticles). Key barriers to clinical translation include targeting heterogeneity, radionuclide availability and supply, chelation chemistry, delivery challenges, dosimetry, toxicity, genetic mutations, drug resistance, regulatory and clinical trial considerations, and clinical implementation. Emerging strategies to overcome these limitations are highlighted. Finally, we discuss future directions for integrating targeted alpha-particle therapy with existing modalities, improved targeting ligands, imaging and dosimetry advancements, expanded indications, and AI integration in the context of ovarian cancer. This scoping review aims to clarify the potential role of targeted alpha-particle therapy in reshaping the treatment landscape for the deadly metastatic ovarian cancer.

## 2. Methods

**Figure 1:**
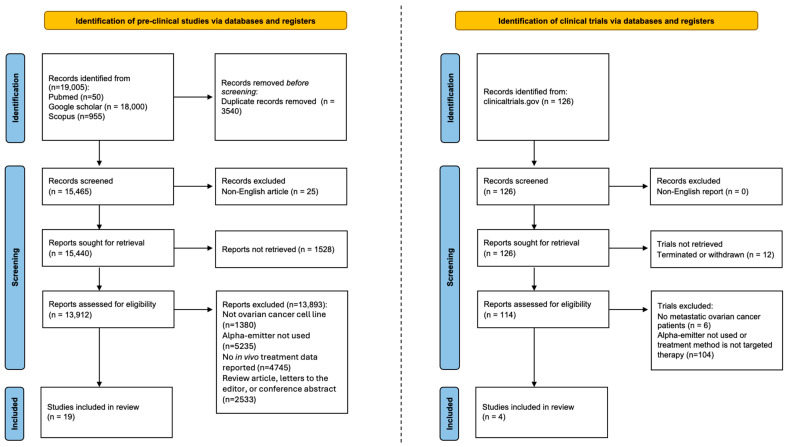
PRISMA 2020 flow diagram of pre-clinical and clinical studies assessing TAT in the context of metastatic ovarian cancer (conducted between September 2025 and November 2025).

This retrospective scoping review includes a detailed methodology description to enhance credibility and transparency, as presented by the PRISMA flow diagram that was performed between September 2025 and November 2025 (Figure 1). We conducted an extensive literature search for clinical trials (clinicaltrials.gov) and pre-clinical studies (PubMed, Scopus, Google Scholar) to ensure comprehensive coverage of relevant advancements. For the clinical studies, the search utilized keywords, including ovarian cancer, radioimmunotherapy, and alpha-particles along with Boolean operators to effectively refine and combine the terms. Four clinical trials were selected that were relevant to our topic after evaluation of their scope. Clinical trials that were terminated or withdrawn were excluded from the screening. For the pre-clinical studies, the search used specific keywords such as ovarian cancer, ovarian carcinoma, radioimmunotherapy, and alpha-particle-emitting, with Boolean operators. Filters were applied to focus on peer-reviewed articles published in English over the past 20 years that used OC mouse models with reported in vivo treatment data. Review articles, letters to the editor, and conference abstracts without original data were excluded. A total of 15,440 publications after removal of duplicates and non-English publications were initially identified with this rigorous process. A total of 19 impactful studies were selected on targeted alpha-particle therapy for ovarian cancer following thorough evaluation of their quality and relevance.

## 3. Biological and Physical Basis of Targeted Alpha-Particle Therapy for Metastatic Ovarian Cancer

### 3.1. Pathophysiology and Current Standard of Care for Ovarian Cancer

Unlike endometrial and cervical cancer, which spread predominantly through the lymphatics to pelvic and para-aortic nodes before hematogenous and lung dissemination [32,33,34], OC favored sites for spread are implantations in/on the omental ‘milky spots.’ These sites have an enrichment of immune cells within the exposed basement membrane [35]. Adipocytes and stromal cells present in these areas provide metabolic substrates and cytokine cues that accelerate tumor growth. Studies using tumor spheroids that incorporate tumor-associated macrophages, cancer-associated fibroblasts, and T cells demonstrate enhanced anoikis resistance and shields from drug exposure, which may correlate with worse overall outcomes [36]. Senescence or transformation of peritoneal mesothelial cells and extracellular matrix remodeling has also been shown to enable more aggressive adhesion and local progression to the peritoneal cavity by cancer cells [37,38,39]. The overall metastatic burden is further increased in malignant ascites, a hallmark of advanced-stage and relapsed OC, acting as an active ecosystem that promotes immune evasion and chemo-resistance and continues the seeding of tumor cells at distant sites [40,41,42]. The clinically challenging features of the metastatic nature of OC caused by microscopic tumor implants on the peritoneal/omental surfaces with limited vascularity further reduce treatment efficacy.

The standard of care for OC is primarily cytoreductive surgery in combination with chemotherapy (systemic and intraperitoneal), but the exact regimen varies depending on the specific circumstances of each patient and the stage of disease [43]. Initial staging of OC is determined using the International Federation of Gynecology and Obstetrics (FIGO) system, which describes the extent and severity of gynecological cancers, including endometrial, cervical, and ovarian cancers [44]. For early-stage OC, the initial treatment is surgery, where the affected ovaries, fallopian tubes, uterus, or other surrounding tissues are removed, depending on the progression and fertility goals [45]. The main prognostic factor is complete cytoreduction, as residual OC tumors have a strong impact on progression-free and overall survival [46], but this is often difficult to achieve if tumors have metastasized. Adjunctive systemic chemotherapy is highly recommended following surgery for high-risk stage 1 and 2a OC, with quality evidence indicating prolonged long-term survival [47,48]. Similarly, the standard treatment of advanced stages of OC with peritoneal metastases has been primary cytoreductive surgery followed by systemic platinum-based chemotherapy. According to the 2025 ASCO guideline updates, significant changes have been made to improve timing and early tumor/germline testing at diagnosis to guide treatment [49]. Primary cytoreductive surgery is now preferred for medically fit patients with a high likelihood of complete cytoreduction at acceptable morbidity, and neoadjuvant chemotherapy is recommended when complete cytoreduction is unlikely or perioperative risk is high. Cisplatin-based HIPEC may also be offered to stage 3 patients treated with neoadjuvant chemotherapy with good performance status and adequate renal function. FDA-approved maintenance options are also offered to patients, including PARP inhibitors with anti-angiogenic therapy (bevacizumab) based on biomarkers and prior therapy. Even with these treatment combinations, a staggering >50% of patients with advanced stages of OC will still develop a recurrence within 3 years following initial treatment [50,51]. External beam radiotherapy has historically been used for whole-abdominal irradiation, but this option fell out of favor due to gastrointestinal/hematopoietic toxicity and does not address diffuse microscopic peritoneal disease [52]. Enhanced permeability and retention (EPR)-based drug delivery systems that use the enhanced permeability and retention effect that passively target drugs to solid tumors underperform for residual OC due to the lack of vascularity in the tumor implantation sites, high interstitial fluid pressure, and heterogeneous structure [53,54,55]. Immunotherapies have recently established roles in other gynecologic cancers, such as metastatic cervical cancer and endometrial cancer, but PD-1/PD-L1 monotherapy response rates are modest (~8–22%) for OC [56,57]. Major challenges with the current standard of care for OC include chemotherapy resistance leading to a high rates of relapse [58,59,60], tumor heterogeneity making it challenging to find a single effective treatment approach [61,62], the complex immune microenvironment of OC that can suppress the immune system for immunotherapy options [63], and the surgical challenges of completely removing all microscopic residual disease in the peritoneal cavity when diagnosed at an advanced stage [64]. This indicates a need for an improved therapeutic strategy that could better address the current shortcomings of advanced-stage OC.

### 3.2. Rationale of Targeted Alpha-Particle Therapy for Ovarian Cancer Treatment

**Figure 2:**
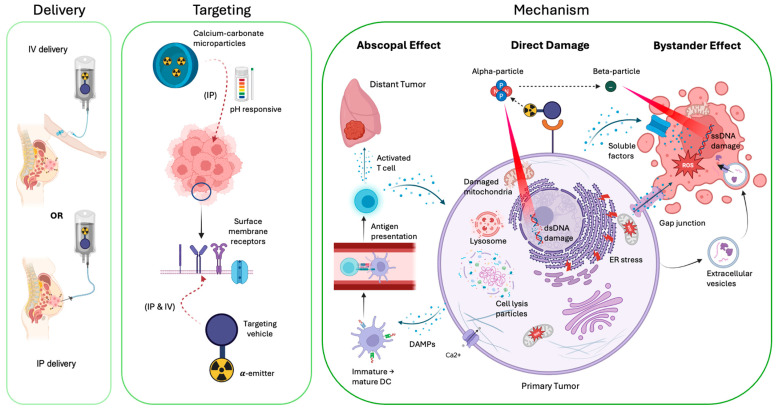
A summary of the delivery (**left**), targeting (**middle**), and biological/physical treatment mechanism (**right**) of TAT in the context of OC. Systemic IV and regional IP delivery are common routes of administration. Targeting of surface membrane receptors (Section 5.1 and Section 5.2) or release of alpha-emitters with pH-responsive calcium carbonate microparticles (Section 5.3) are the methods of targeting OC tumors. Direct damage, abscopal effect, and bystander effect are the three mechanisms of alpha-particle cellular damage.

Targeted alpha-particle therapy (TAT) is an emerging cancer treatment that uses an alpha-particle-emitting radionuclide linked to antibodies or other targeting molecules to specifically target and destroy cancer cells [65]. Figure 2 provides an overview of TAT delivery, targeting, and biological/physical treatment mechanisms in the context of OC. The physical advantage of alpha-particles is their ability to deliver very high linear energy transfer (LET) of ~50–230 keV/μm over a short path length of ~50–100 μm (~5–10 cells in diameter) [66]. This leads to dense, difficult-to-repair dsDNA damage with a low dependence on oxygenation status or active cell phase cycle and reduces damage to neighboring healthy cells compared to beta-particles that primarily produce single-stranded DNA damage [23,67]. The hypoxia tolerance property of TAT can provide significant advantages in treating hypoxic radiation-resistant refractory tumors with poorly vascularized clusters [68]. Intraperitoneal (IP) administration of TAT is particularly attractive, with the ability to irradiate the layer where residual disease persists and the specific targeting of radio-labeled antibodies, thereby enhancing treatment efficacy post-cytoreductive surgery. In addition to the direct dsDNA damage, alpha-particles generate reactive oxygen species when they interact with water to a higher degree than other radiation types, resulting in damage to proteins and phospholipids associated with the cell membrane, mitochondria, endoplasmic reticulum, and lysosomes [69,70,71].

Apart from direct physical cytotoxicity, TAT (similar to other radiation types) can also cause biological damage through bystander and abscopal non-targeted effects. The bystander effect occurs when irradiated cells convey damage and increase the radiosensitivity of neighboring non-irradiated cells through gap junction channels [72,73,74], soluble mediators (reactive species, cytokines, ionic signaling mediators) [75,76,77], extracellular vesicles [78,79], or ssDNA damage from accompanying beta-particles with a range of several mm [80]. Interestingly, the bystander effect diminishes at higher alpha-particle radiation doses, providing a strong rationale for using a lower fractionated therapy approach [81]. Additionally, the abscopal effect modulates the immune system and can trigger a long-term anti-tumoral response, further eradicating any remaining tumors that were not directly targeted [82]. Studies have found that radiation increases inflammation in tumors by releasing damage-associated molecular patterns (DAMPs), thereby activating the nuclear factor κB and type 1 interferon response pathways to induce the expression of pro-inflammatory cytokines [83,84]. This facilitates dendritic maturation and cross-presentation of tumor antigens to prime tumor-specific T cell responses, along with enhancing T cell infiltration into tumors to trigger immunological cell death. This therapeutic effect is further enhanced with alpha-emitters’ reduction in immunosuppressive regulatory T cells [85]. Additionally, systemic and continued anti-tumor response of TAT has been observed with the release of MHC-I molecules from tumor cells, promoting the formation of effector memory T cells [82,86]. Although the molecular and cellular effects of alpha-particles on tumor cells are only beginning to be understood, given the limited molecular studies, an improved understanding of the treatment mechanisms would be crucial for the discovery of new targets, implementation of combination strategies, and optimization of patient selection and treatment scheduling.

These properties of alpha-particles have led to encouraging treatment efficacy across multiple malignancies with characteristics similar to OC, such as micro-metastases and treatment-refractory disease. In neuroendocrine tumors, ^212^Pb-DOTAMTATE and ^225^Ac-DOTATATE have demonstrated meaningful treatment response and stabilization in early-phase clinical trials, resulting in improved Peptide Receptor Radionuclide Therapy (PRRT) compared to beta-emitters (i.e., ^117^Lu-DOTATATE), with safety profiles consistent with the underlying disease and similar toxicities as beta PPRTs [27,87]. This highlights the ability of alpha-emitters to overcome radiation resistance from previous radiation treatment. In metastatic castration-resistant prostate cancer (mCRPC), ^223^Ra dichloride TATs have demonstrated significant survival benefit in various clinical trials as monotherapy or combination therapies [88,89,90]. In 2013, this led to the first FDA regulatory approval of TAT, Xofigo^®^, for treating mCRPC with symptomatic bone metastases without known visceral metastases and the proof of concept of TAT in solid tumors. The PEACE-3 trial studied Xofigo^®^ combined with enzalutamide, which found that the combination significantly improved radiological progression-free survival compared to enzalutamide alone [88]. Recently, prostate-specific membrane antigen (PSMA) TAT, including ^225^Ac-PSMA-617, has produced high prostate-specific antigen response rates and tumor control in heavily pre-treated mCRPC patients, including those with radiation resistance from beta-emitting radioligand therapies [91]. Completed early phase 1 and 2 TAT clinical studies for hematological malignancies demonstrated objective treatment responses and safety as a monotherapy or combined with low-dose chemotherapy [92]. TAT could be useful in intensifying anti-leukemia therapy prior to hematopoietic cell transplant. Other early-phase TAT clinical studies that are actively recruiting include that of advanced melanoma (NTC05655312) in combination with approved immunotherapies. These promising clinical trial findings from various advanced malignancies collectively provide a strong rationale for TAT application in metastatic, treatment-resistant OC [93]. TAT is currently being investigated in both pre-clinical studies and clinical trials for various solid tumors, including advanced OC, using a variety of alpha-emitting radioisotopes (e.g., ^225^Ac, ^211^At, ^212^Pb, ^223^Ra, and ^213^Bi), which will be discussed in the following sections.

## 4. Pre-Clinical Evidence of TAT in OC

Over the past two decades, pre-clinical investigations of TAT for metastatic OC have established convincing translational rationales and steadily shaped the conceptual foundation for the IP delivery of high LET alpha-emitter-labeled antibodies or peptide vectors across multiple metastatic OC models (i.e., epithelial OC, post-operative peritoneal carcinomatoses, and xenografted micro-metastases). As previously mentioned, these studies have emerged from the core challenge that OC most often occurs as diffuse, microscopic tumors distributed throughout the peritoneal cavity, where conventional therapy struggles to deliver curative doses. A diverse array of radionuclides (i.e., ^211^At, ^213^Bi, ^225^Ac, ^212^Pb, ^214^Pb) has been explored. The relevant studies, as outlined in Table 2, have consistently demonstrated that targeting clinically relevant antigens (i.e., NaPi2b, folate receptor-α, B7-H3, syndecan-1, HER2, etc.) can achieve high tumor-free fractions (TFFs), eradicate microscopic peritoneal implants, and demonstrate favorable tolerability across a range of doses with limited systemic toxicities.

Three dominant therapeutic TAT paradigms (single-dosing, fractionated, and pre-targeted approaches) have been used to treat microscopic peritoneal OC metastases, each offering distinct advantages. The single-dose IP TAT has consistently demonstrated the most robust and reproducible anti-tumor efficacy, reliably suppressed ascites formation, and prolonged survival in micro-metastatic disease mouse models across a variety of targets (folate receptor-α, MUC1, CD138, NaPi2b, B7-H3, PTK7, HER2, TAG-72, and MUC16). Toxicity was generally mild, predominantly showing transient cytopenias or low-grade renal tubular changes at higher activity levels or with longer-lived isotopes (e.g., ^225^Ac). OC studies also explored fractionated dosing strategies to mitigate toxicity and improve tolerability at higher cumulative absorbed doses or with longer-lived isotopes. Fractionation showed comparable anti-tumor efficacy to single dosing while reducing hematologic suppression and improving weight recovery in NaPi2b and TAG-72 targeted studies. Other NaPi2b-targeted studies have shown improved anti-tumor efficacy and ascites elimination when sufficient cumulative activity was delivered, suggesting that fractionation may be most advantageous when toxicity is dose-limiting. The pre-targeted approach is a more complex strategy aimed at separating tumor targeting from radionuclide delivery to improve tumor:normal tissue uptake. In HER2-targeted OC models, pre-targeting significantly prolonged survival and suppressed tumor burden with mild renal histopathologic changes. Compared to single-dosing, pre-targeting of NaPi2b reduced ascites and macroscopic tumor burden at comparable or lower activities. Pre-targeting may be a promising approach when antigen expression is more heterogeneous or when antibody penetration is limited. Taken together, single-dose TAT remains the most effective and robust approach for disseminated peritoneal OC, while fractionated and pre-targeting approaches offer effective alternatives to optimize the safety and therapeutic window. Despite the inherent limitations of immunodeficient, xenograft models used in the studies, these findings collectively highlight the unique suitability of TAT for the improved treatment of disseminated peritoneal metastases. This provides a foundation for early-phase clinical trial translation for patients with advanced or recurrent OC. We further explore the details of the nine major pre-clinical TAT studies associated with OC in the Supplementary Materials [94,95,96,97,98,99,100,101,102,103,104,105,106,107,108,109,110,111,112,113,114,115].

**Table 2:** Summary of pre-clinical TAT OC studies.

Target	Vector	Alpha-Emitter	Model	Route	Dose/Timing	Reported Toxicity	Primary Treatment Outcome
FR-α	MOv18 [94]	^211^At	Female nude mice, IP inoculation with OVCAR-3; therapy delivered either when the disease was microscopic or when macroscopic with ascites.	IP	Microscopic disease cohort: single ~450–555 kBq. Macroscopic/ascites cohort: single ~377–389 kBq. Controls: untreated	No specific toxicity or treatment-related deaths were reported in the microscopic disease and advanced-disease/ascites cohort.	Microscopic disease: median survival 213 days with ^211^At-MOv18 vs. 138 days in untreated controls; tumor-free fraction (TFF) = 33% of treated mice at 7 months. Macroscopic/ascites disease: ~377–389 kBq delayed ascites formation (palliative).
	Farletuzumab [95]	^211^At	Nude mice bearing IP-disseminated OVCAR-3 ovarian cancer.	IP	Single IP injection: ~170 kBq/mL. Controls: ^211^At rituximab (unspecific) ~170 kBq/mL, unlabeled farletuzumab, and PBS.	Thyroid uptake was observed when no blocking agent was used.	TFF at endpoint: ^211^At-farletuzumab ~91%, PBS 12%, unlabeled farletuzumab 9%, and unspecific ^211^At-rituximab 14%; 6–10× higher anti-tumor effect for ^211^At-farletuzumab compared with controls.
	MOv18 [96]	^211^At	Female Balb/c nu/nu nude mice inoculation with OVCAR-3 cell IP. Therapy was initiated 2 weeks post-inoculation (microscopic disease stage).	IP	Single IP injection: ~300–400 kBq per mouse (2 weeks after tumor inoculation). Controls: PBS, unlabeled MOv18, or ^211^At-unspecific mAb (C242).	No obvious side effects reported at therapeutic dosing (~300–400 kBq); WBC depression was insignificant.	TFF: IP ^211^At-MOv18 at ~400 kBq 93%, IP-unlabeled MOv18 10%, non-specific control 25%, and PBS 0%; all surviving animals were free from macroscopic tumors; durable complete responses in ~1/3 of mice at 7 months; ascites prevented in almost all treated animals.
MUC1	C595 [97]	^213^Bi	Nude mice bearing OVCAR-3 ovarian cancer cells as an intraperitoneal ascites model.	IP	Single IP injection: 355, 710, or 1065 MBq/kg (9 days post-inoculation). Controls: not specified in the dosing/timing cell.	Minimal toxicity observed, no leukocyte depression at 90 days, mild renal tubular changes/mild radiation nephropathy at 1065 MBq/kg.	Single 355 MBq/kg IP prolonged survival by ~25 days vs. control in the ascites model.
CD138	B-B4 [98]	^213^Bi	Nu/nu nude mice, inoculated IP with SHIN-3-Luc cells. Therapy was administered 3 days post-inoculation to mimic post-operative minimal residual disease.		Single IP injection: 7.4 MBq or 11.1 MBq (3 days post-inoculation). Controls: HIPEC (oxaliplatin 5 mg/kg, 42 °C, 90 min) and HIPEC + alpha-RIT.	Transient, mild hematologic effects with insignificant decreases in WBC and platelets; no acute blood toxicity at 7.4–11.1 MBq ^213^; transient weight loss recovered by day 7.	Median survival: control 68 d; HIPEC 37.5 d; HIPEC + α-RIT 75.5 d; ^213^Bi-B-B4 7.4 MBq > 90 d (7/11 alive at 90 d); ^213^Bi-B-B4 11.1 MBq > 90 d (5/7 alive); ascites and tumor burden markedly reduced in α-RIT groups compared with controls/HIPEC.
B7-H3 (CD276)	376.96 [99]	^212^Pb ^212^Bi	ES-2 (ascites) IP xenografts (athymic mice), A2780cp20 (nodular) IP xenografts (athymic mice).	IP	Single IP injection: 0.17, 0.35, 0.51 MBq for ES-2 (day 4 tumors) and 0.35, 0.53, 0.70 MBq ± weekly carboplatin 50 mg/kg (carboplatin given 1 day before ^212^Pb-376.96) for A2780cp20 (day 10 tumors). Controls: ^212^Pb-F3-C25 and untreated.	Transient weight loss recovered by 2–4 weeks; no blood/organ pathology.	Treated groups with ^212^Pb-376.96 (alone or with carboplatin) survived 2–3× longer than those treated with ^212^Pb-F3-C25 or untreated controls.
HER2	2Rs15d single-domain antibody fragment [100]	^213^Bi	Female athymic nude mice, SKOV-3 IP (luciferase^+^).	IV	Study 1 (fractionated I.V. injection on days 7.8. and 24): 1.01 ± 0.05 MBq × 3 or 2.12 ± 0.11 MBq × 3; vehicle control. Study 2 (fractions on days 7, 9, 11): 1.03 ± 0.03 MBq × 3 or 0.5 ± 0.08 MBq × 3; same two regimens + trastuzumab (loading 7.5 mg/kg, then 3.5 mg/kg maintenance); trastuzumab alone; vehicle.	Dose-limiting renal toxicity (≥2 MBq) caused kidney histopathology (tubular dilatation/degeneration/necrosis and medullary interstitial fibrosis + mononuclear infiltrates); >20% weight loss in high-dose and repeat-dose groups; spleen hemosiderin in red pulp with increasing activity.	Study 1: 1 MBq × 3 median survival 68 d vs. 56 d control; 2 MBq × 3 not significantly better than control. Study 2: 0.5 MBq × 3 mean overall survival 80 d vs. control 53 d, 1 MBq × 3 median 67 d vs. 53 d control, 1 MBq × 3 + trastuzumab median 145 d vs. 109 d trastuzumab alone, and 0.5 MBq × 3 + trastuzumab median 140.5 d (increased 28% vs. trastuzumab alone). BLI showed delayed tumor growth in all treated groups.
	Pre-targeted. 1: Bispecific antibody (anti-HER2). 2: ^225^Ac-Proteus-DOTA [101]	^225^Ac	Female athymic nude mice, injected IP with SKOV3-luc cells.	IP	One or two cycles of PRIT; 5 mg/kg bsAb (day 0) per cycle → 24 h later 37 kBq ^225^Ac-Proteus-DOTA (day 1). Day 15 repeat for the 2nd cycle group. Control: irrelevant bispecific antibody and radiohapten.	Well tolerated, no significant weight loss, minimal-mild renal tubular changes, and hematology within reference limits.	2-cycle PRIT group: 85% (17/20) alive at day 133 vs. 37% (10/27) in control; 15/15 PRIT-treated mice had no viable carcinoma.
NaPi2b (SLC34A2)	MX35 F(ab′)_2_ [102]	^211^At	Female nude mice, IP inoculated with OVCAR-3 human ovarian cancer cells.	IP	Single IP injection: 25, 50, 100, 200 kBq (4 weeks post-inoculation). Non-specific control: 100 kBq ^211^At-rituximab F(ab’)2.	Toxicity not directly assessed/reported, toxic limit discussed only in reference to prior myelotoxicity studies.	TFF at 4 weeks: 25 kBq achieved 25%, 50 kBq 22%, 100 kBq 50%, 200 kBq 61%.
	MX35 F(ab′)_2_ [103]	^211^At	Female nude BALB/c nu/nu mice; IP injected OVCAR-3 cells. Therapy was given 3 weeks post-inoculation.	IP	Single IP treatment: ~400, 800, or 1200 kBq; therapy was given 3 weeks post-inoculation. Controls: unlabeled MX35, PBS, ~400 kBq ^211^At -MOv18.	Well-tolerated; treated mice had lower body weights than controls at 2 months.	^211^At-MX35 (all dose groups combined): 3/25 ascites, 0/25 macroscopic tumors, 8/25 microscopic tumor growth. ^211^At-MOv18 (~400 kBq): 0/10 ascites; 0/10 macroscopic tumors; 3/10 microscopic tumor growth. Controls (unlabeled MX35 or PBS): 18/18 ascites, 6/9 macroscopic tumors, all had microscopic tumor growth.
	MX35 F(ab′)_2_ [104]	^211^At	5-week-old nude mice inoculated intraperitoneally with OVCAR-3 cells; groups treated at different tumor ages to span micro- to larger clusters.	IP	Single IP dose ~400 kBq 211At-MX35 F(ab’)2 (specific) delivered 1, 3, 4, 5, or 7 weeks after tumor cell inoculation. Controls: ~400 kBq 211At-rituximab F(ab’)2 (non-specific) and unlabeled rituximab.	Toxicity not directly assessed/reported; potential renal toxicity warrants further study.	TFF by treatment timing: ^211^At-MX35: 95%, 68%, 58%, 47%, 26% when treated at 1, 3, 4, 5, 7 weeks; ^211^At -rituximab (non-specific): 100%, 80%, 20%, 20%, 0% at 1, 3, 4, 5, 7 weeks.
	MX35 F(ab′)_2_ [105]	^213^Bi and ^211^At	Female BALB/c nu/nu nude mice, 6–8 weeks old. IP inoculation OVCAR-3 cells. Therapy given at 2 or 4 weeks post-inoculation.	IP	2 weeks post-inoculation: 0.35–0.54 MBq ^211^At -MX35 or 2.55–2.95 MBq Bi-213-MX35. 4 weeks post-inoculation: 0.42 MBq 211At-MX35 or 2.51–3.08 MBq Bi-213-MX35. Controls: untreated control.	Transient hematologic toxicity with decreased WBC at day 5 (~10% for ^213^Bi-MX35; ~45% for ^211^At-MX35) and recovery to baseline by day 14; no weight loss.	TFF: ^211^At-MX35 90% and ^213^Bi-MX35 60%; efficacy fell for larger/older tumors (TFF 0.25 at 4 weeks).
	MX35 F(ab′)_2_ [106]	^211^At	Female Balb/c nu/nu nude mice inoculated IP with OVCAR-3 cells. Therapy given 4 weeks post-inoculation.	IP	400 kBq 211At-MX35 F(ab’)_2_ per injection delivered once or weekly up to 6 times. Controls: unlabeled MX35 F(ab’)2.	No hematological or organ toxicity; controls weighed more due to ascites.	TFF (no macro-/micro-tumor and no ascites): 0.17, 0.11, 0.39, 0.44, 0.44, 0.67 for 1, 2, 3, 4, 5, 6 weekly injections; ascites fell from 15/18 (one dose) to 0/18 (5–6 doses); unlabeled control TFF = 0.
	MX35 F(ab′)_2_ [107]	^211^At	BALB/c nu/nu nude mice IP-inoculated intraperitoneally with OVCAR-3 cells at age 5 weeks. Tumors allowed to grow for 4 weeks before treatment began.	IP	Single dosing: 50, 400, 800 kBq. Fractionated dosing (day 1, 4, 8): 3 × 17, 3 × 133, 3 × 267 kBq. Control: untreated control.	Hematologic toxicity (myelotoxicity/leukopenia) observed; fractionated regimen reduced myelotoxicity vs. single dose and delayed WBC nadir.	TFF: 50 kBq 17%, 3 × 17 kBq 22%, 400 kBq 39%, 3 × 133 kBq 28%, 800 kBq 56%, 3 × 267 kBq 41%, unlabeled MX35 F(ab’)2 controls 0% (high rates of macroscopic tumors, microscopic disease, and ascites).
	Pre-targeted. 1: avidin-MX35. 2: ^211^At biotinylated, succinylated poly-L-lysine [108]	^211^At	Female BALB/c nu/nu nude mice; OVCAR-3 cells IP-inoculated. Necropsy/response assessment occurred 8 weeks after therapy.	IP	PRIT 1 (lower activity): avidin-MX35 (25 µg) → 24 h → ^211^At-B-PLsuc 1.0 MBq/0.4 µg (0.75 mL PBS). PRIT 2 (higher activity): avidin-MX35 (25 µg) → 24 h → ^211^At-B-PLsuc 1.5 MBq/0.6 µg (0.75 mL PBS). RIT: 211At-MX35 0.9 MBq/4 µg (0.75 mL PBS). Controls: PBS.	Acute, reversible myelotoxicity with significant WBC and platelet suppression at day 5 and recovery by ~day 21.	TFF (no ascites, no macro-/microscopic tumors): PRIT 1 35%, PRIT 2 45%, RIT 0.9 MBq 45%, and control 0%. Ascites at necropsy: RIT 40%, PRIT 1 15%, PRIT 2 0%. Tumors > 1 mm: PRIT 1 and RIT 40%, PRIT 2 15%.
TAG-72	CC49 [111]	^225^Ac	Female athymic nude mice with subcutaneous OVCAR-3 xenografts (~100 mm^3^ at therapy start).	IP	Single-dose cohorts: 1.85, 3.7, or 7.4 kBq per mouse. Fractionated regimen: 1.85 kBq initial dose + 0.74 kBq weekly × 5 (total 5.55 kBq). Controls: vehicle, unlabeled huCC49, or ^225^Ac-IgG.	Dose-dependent weight loss < 20%; fractionated was better tolerated than a single 7.4 kBq dose (less weight loss, reduced acute toxicity); targeted 7.4 kBq had better tolerability than untargeted.	Single-dose efficacy (7.4 kBq): tumor regression in most mice; median survival > 120 days vs. ~30–40 d in controls. Fractionated regimen: 5.55 kBq split dosing extended survival to ~100 d vs. ~30 d controls.
MUC16	AR9.6 [112]	^225^Ac	Female athymic nude (Nu/Nu) mice subcutaneous OVCAR3 xenografts; initial injection of 10 × 10^6^ cells, followed by 5 × 10^6^ cells one week later; tumors allowed to reach ~200–600 mm^3^ before treatment.	IV	RIT: single-dose 0.037 MBq PRIT: AR9.6-TCO followed by 0.148 MBq 72 h later. Controls: saline; IgG RIT control: 0.037 MBq, single dose; IgG PRIT control: IgG-TCO followed by 0.148 MBq AR9.6 72 h later.	Transient hematologic toxicity (WBC/RBC/platelets recovered by 2–4 weeks); early weight loss (~5–10% with recovery by day 14), with 1 PRIT mouse euthanized for >20% loss); mild-moderate renal tubulonephropathy; moderate ovarian cortical atrophy/follicular loss.	Median survival: AR9.6 RIT ~80 days, AR9.6 PRIT ~80 days, IgG~60 days, and saline controls ~25 days; complete tumor responses without recurrence in surviving mice from both treatment groups.
PTK7	chOI-1 [114]	^212^Pb	Female BALB/cOlaHsd-Foxn1^n^_u_/^n^_u_ nude mice, 6–8 weeks old, IP xenograft model A2780 cells. Treatment initiated at day 18 post-inoculation.	IP	Single IP injection: 458 kBq ^212^Pb-TCMC-chOI-1 (~50 MBq/mg) on day 18 post-tumor inoculation. Controls: free ^212^Pb (470 kBq), unlabeled chOI-1 (10 µg), and vehicle formulation buffer.	Mild transient weight loss stabilized by day 5; no comprehensive toxicity assessment performed (no hematology/organ pathology).	Median survival: 42 days for ^212^Pb-TCMC-chOI-1 and 22–25.5 days for all control groups; reduced abdominal distension and tumor burden compared with controls.
	mOI-1 [115]	^212^Pb	Female athymic nude mice IP xenograft model: SKOV-3-luc cells. Treatment was initiated 3 days post-inoculation to model early peritoneal disease.	IP and IV	Single IP injection: 180 kBq and 405 kBq ^212^Pb-TCMC-chOI-1 (3 days post-inoculation). Controls: saline, unlabeled chOI-1, ^212^Pb-TCMC-hIgG (211 or 384 kBq).	Toxicity not comprehensively assessed; need blood counts and histopathology in future studies.	TFF: 180 kBq ^212^Pb-TCMC-chOI-1 100%. 405 kBq ^212^Pb-TCMC-chOI-1 87.5% (7/8), ^212^Pb-TCMC-hIgG controls 25%, non-radioactive controls 0%. TFF = total IP tumor weight ≤ 0.020 g at endpoint.

## 5. Clinical Evidence of TAT in OC

An increasing number of commercial pharmaceutical companies and investigator-initiated trials have started to explore the use of TAT for a variety of cancer types, but those focused on OC patients remain limited. This section provides an overview of both the completed and recruiting/active clinical trials. A variety of targets for OC have been explored, including HER2 and NaPi2b (SLC34A2) proteins. A handful of these trials have reported promising preliminary data, leading to potential upcoming phase II trials. These TAT OC clinical trials are summarized in Table 3, highlighting the type of alpha-particle, target, indication, dosing schema, phase/trial status, and primary outcomes.

### 5.1. ^211^At-MX35 F(ab’)2 Targeting NaPi2b

In the first clinical trial, a Swedish group performed a single-center phase 1 study evaluating intraperitoneal TAT for OC using ^211^At MX35 F(ab’)2 following extensive pre-clinical work (NCT04461457). This approach uses the high LET and short tissue range of ^211^At conjugated to MX35 F(ab’)2 antibody fragment targeting NaPi2b, a surface antigen specifically expressed in >90% of human epithelial OC for the eradication of microscopic peritoneal residual disease [102,116]. Women with recurrent epithelial OC who had achieved complete or near-complete response to second-line chemotherapy and confirmed to have no macroscopic tumor or major adhesions via laparoscopy were selected for the study [117]. Patients received escalating doses of intraperitoneal ^211^At-MX35 F(ab’)2 ranging from 20–215 MBq/L. The overall median survival was 35 months, with a 1-, 2-, 5-, and 10-year survival of 100%, 83%, 50%, and 25%, respectively. Four of these patients had a survival of more than 6 years, with one of these having no relapse. Treatment was well tolerated, with toxicity limited to transient, low-grade procedural symptoms. Hematologic, renal, and late radiation-induced toxicities were not observed. These survival rates are drastically improved compared to the 13–25 months survival of OC patients after relapse, depending on platinum resistance [118]. Nine of the patients underwent dosimetry and pharmacokinetic testing, where 22.4–101 MBq/L of ^211^At-MX35 F(ab’)2 was infused with a peritoneal catheter. γ-Camera and SPECT scans at 6 h post-infusion were acquired. Potassium perchlorate effectively suppressed thyroid uptake from 127 ± 63% initial activity concentration (IC) to <20% IC [119]. Following application of tissue weighting factors, the largest contributors to the effective dose were the lungs, stomach, and urinary bladder. Organ-equivalent doses were less than 10% of the estimated dose tolerance when administering 100 MBq/L of ^211^At-MX35 F(ab’)2 [120]. These sets of data demonstrated that it was possible to achieve therapeutic absorbed doses in microscopic tumor clusters with low systemic organ doses. This clinical trial was completed in July 2020, and these results provide the first human evidence that intraperitoneal TAT using ^211^At-MX35 F(ab’)2 can safely and effectively treat microscopic peritoneal metastases for OC patients. Although the next steps of this therapy have not yet been announced, these results support the continued development of TAT for OC patients at high risk of peritoneal recurrence.

### 5.2. ^212^Pb-TCMC-Trastuzumab Targeting HER2

Another first-in-human phase 1 clinical trial was conducted by Meredith et al. for IP ^212^Pb-TCMC-trastuzumab, evaluating safety, biodistribution, and efficacy in patients with relapsed HER2-expressing carcinomatosis who have failed standard therapy, where 15/16 cases were due to OC (NCT01384253). Pre-clinical studies have shown that trastuzumab antibody selectively binds to SKOV3 (K_d_ = 2 ± 1 nM) and OVCAR3 (K_d_ = 3 ± 1 nM) OC cell lines, while the high-LET alpha-emissions of ^212^Pb enable potent, short-range cytotoxicity ideal for microscopic residual disease [121]. A standard 3 + 3 dose escalation phase 1 design was IP-treated over escalating five dose levels, 7.4–27.4 MBq/m2, less than 4 h after giving 4 mg/kg of IV trastuzumab antibody [122]. Toxicity was consistently mild, limited to transient grade 1 abnormalities. Even at 1-year follow-up, no dose-limiting hematologic, renal, hepatic, or cardiac toxicity was noted. Standard CT imaging showed reduced tumor size, but no patient met criteria for a partial response to Response Evaluation Criteria in Solid Tumors (RECIST) criteria at 6 weeks. A pharmacokinetic and imaging study was additionally performed with 0.2 mCi/m2 IP ^212^Pb-TCMC-trastuzumab following 4 mg/kg of IV trastuzumab antibody [123]. The results demonstrated favorable pharmacokinetics with prolonged peritoneal cavity retention, minimal systemic redistribution, <6% urinary excretion, and no appreciable uptake in major organs consistent with pre-clinical studies. In terms of the tumor markers assessed, TAG-72 had a pattern comparable to that of tumor growth changes, but no associations were found with CEA, CA125, HE-4, SAA, or IL-6 [124]. This study was completed in July 2016 and established the excellent safety and feasibility of up to 27.4 MBq/m^2^ ^212^Pb-TCMC-trastuzumab for HER2-expressing carcinomatosis, including OC. This supports the further application at higher doses where TAT is most likely to be effective, such as for microscopic or minimal residual intraperitoneal disease following cytoreduction/chemotherapy, or in combination with radiosensitizers. The next steps would be to perform dose escalation and evaluate optimized timing and synergy with other treatment modalities to determine improved treatment efficacy.

### 5.3. Radspherin^®^ (^224^Ra Calcium Carbonate Microparticles)

The most recent TAT application for OC is Radspherin^®^, developed by Oncoinvent, an IP suspension of biodegradable calcium carbonate microparticles labeled with ^224^Ra (t1/2 = 3.6 days). Biodegradable calcium carbonate microparticles (Figure 2) are known to release drugs, including radiopharmaceuticals, into tumors through the pH-responsive acidic environment and the enhanced permeability and retention effect via leaky tumor vasculature [125]. Radspherin^®^ is currently being evaluated in two early-phase clinical trials (NCT03732768, phase 1, and NCT06504147, phase 2) to target microscopic residual peritoneal disease following cytoreductive surgery for platinum-sensitive recurrent epithelial OC. In the first-in-human, dose escalation phase 1 study, platinum-sensitive OC patients were IP-injected two days after surgery at 1, 2, 4, and 7 MBq dose escalation. The initial toxicity results demonstrated that the dose-limiting toxicity was not observed across all dose levels, with only one serious adverse event related to administration [126]. Although not yet published as a peer-reviewed manuscript, Oncoinvent reports that only one of the 10 patients receiving the highest IP dose had peritoneal recurrence at 24 months follow-up [127]. These rates are drastically lower than ~55–60% disease recurrence of similar patients receiving the best standard of care [128,129,130]. The ongoing phase 2 clinical trial (2031 expected completion date) of Radspherin^®^ is expanding enrollment to include patients undergoing primary or interval debulking surgery to refine dosing, safety monitoring, and exploratory efficacy endpoints (e.g., progression-free survival and peritoneal recurrence patterns). The most recent outcomes of the phase 1 trial establish Radspherin^®^ as a promising IP TAT platform with a strong safety profile and potential to improve local control of OC.

## 6. Challenges

TAT has shown considerable promise in treating OC; however, several biological, technical, and logistical challenges must be addressed before this can be routinely implemented in clinical practice. One limitation is the heterogeneous expression of target antigen across OC subtypes, between patients, and even within individual lesions [131,132]. Some of the key markers from the previously mentioned sections have been shown to exhibit variable expression between primary and metastatic lesions and may be downregulated following chemotherapy or immune challenges [133,134,135]. This limits uniform TAT uptake, which results in intratumoral ‘cold regions’ that may be shielded from energy deposition and reduce therapeutic efficacy [136]. This uneven distribution is further exacerbated by the restricted penetration of large antibody vectors that have difficulty penetrating bulky tumor nodules within a fibrotic stromal environment, particularly evident following surgery [137,138]. Clinically, this would make treatment response difficult to predict as target expression can vary between patients and within different tumor cell populations or microenvironments in the same patient.

**Figure 3:**
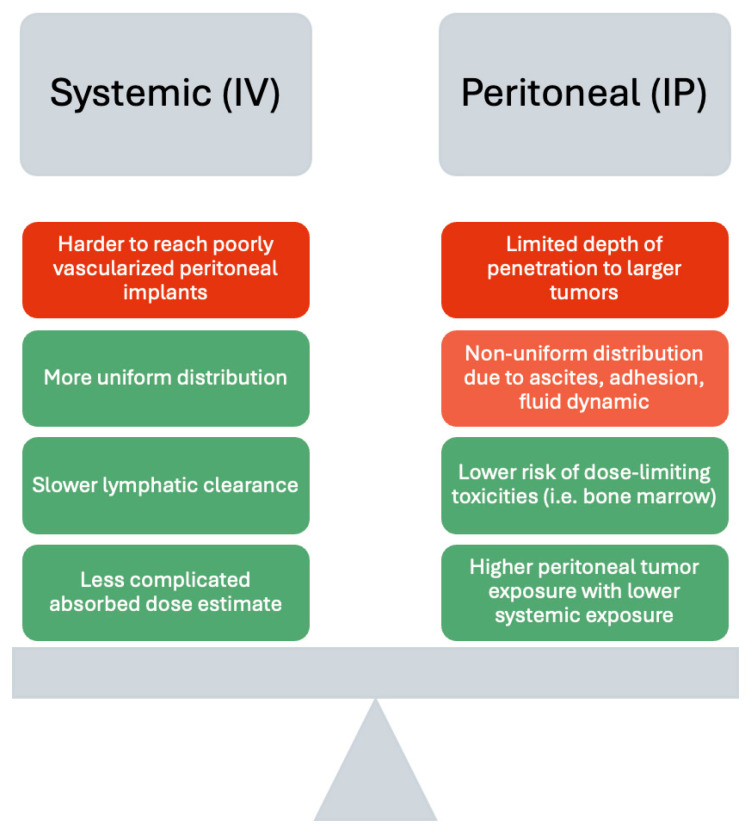
Comparison of systemic (IV) and localized peritoneal (IP) administration of TAT. Green indicates benefits and red indicates limitations of the delivery method.

Another major challenge lies in the limited delivery routes available for TAT in OC (Figure 3). IP administration remains the commonly employed approach in the pre-clinical studies and clinical trials, given the poor vascularity of metastasis. Despite the theoretical advantage of coating the peritoneal surface, achieving homogenous distribution is difficult due to post-operative adhesions, ascites development, anatomic variability, and complex peritoneal fluid dynamics [139,140]. Repeated administrations of lower-energy gamma radiation may promote aggressive adhesion formation, fibrosis, or chronic inflammation within the peritoneal cavity and select for more aggressive cancer phenotypes, further exacerbating the uneven distribution over time [141]. The rapid lymphatic clearance of drugs delivered into the peritoneal cavity may also shorten residence time and reduce the effective tumor exposure [142,143]. In vitro binding assays and in vivo biodistribution data from previously listed pre-clinical studies have consistently demonstrated rapid target engagement and high tumor uptake following IP administration in OC xenograft models. However, these favorable kinetics may not always clinically translate to predictable dose distributions, and this variability complicates treatment planning, dose escalation, and outcome reproducibility. On the other hand, systemic delivery of TAT may overcome distribution challenges, but toxicity may be more severe compared with localized peritoneal delivery [144]. Due to the high sensitivity of rapidly dividing cells to radiation, the bone marrow is usually one of the dose-limiting organs, causing suppression manifesting as anemia, leukopenia, and thrombocytopenia with corresponding risks of infection or bleeding [145]. TAT can also damage immune cell populations, particularly in heavily pre-treated patients, leading to immunosuppression, which may reduce tumor immune surveillance and limit the efficacy of subsequent immunotherapies [146]. Gastrointestinal irritation and renal toxicities are other frequently reported adverse events more commonly associated with the administration of targeted radionuclide therapy [147]. Furthermore, the inherent heterogeneous distribution of IP administration using alpha-emitters complicates accurate absorbed dose estimates and renders organ-level dosimetry unreliable. Due to the extremely short path length and highly localized energy of alpha-emitters, accurate measurements require micro-dosimetry modeling at the cellular level that is largely inaccessible in routine clinical practice [148]. Areas that need to be addressed to reduce the uncertainty of dose planning and toxicity prediction include the lack of reliable imaging surrogates, uncertainty in intratumoral ligand distribution, and the stochastic nature of alpha-particle interactions.

At the physical level, the recoil effect associated with alpha decay is another limitation of TAT. Recoil energy is produced when the alpha-emission exceeds the threshold to break the chelator–radionuclide bonds, allowing daughter isotopes to dissociate and be redistributed systemically [26,149]. This is specifically relevant to ^225^Ac and ^212^Pb, given the higher number of daughter isotopes. Even if the parent compounds are accurately delivered to the tumors, these free daughter nuclides could potentially accumulate in radiosensitive organs (i.e., kidney, liver, bone, thyroid) and increase the risk of off-target toxicities. This emphasizes the need to improve chelation chemistry and retention strategies. Even though alpha-particles have a very short range, the radiation-induced bystander effect is another complication that can increase toxicity when unirradiated cells near the target develop DNA damage and instability due to signals from irradiated cells (i.e., reactive oxygen species and cytokines) [150,151]. This is particularly relevant for advanced-stage OC, where disseminated micro-metastases are distributed within the peritoneal lining. Studies would need to be performed to assess if the bystander effect can extend to regions within the larger tumor clusters that are unable to be accessed by initial targeting with substantial cytotoxicity for eradication.

Intrinsic resistance mechanisms of cancer cells are another challenge that may blunt the effectiveness of targeted radiation therapies, which may limit the anti-tumoral efficacy and lead to treatment resistance or disease recurrence [152]. TAT can also induce adaptive resistance when tumor cells adapt to the damage through rewiring of DNA damage response pathways and selection of tumor subpopulations with enhanced activation of non-homologous end joining, altered cell cycle checkpoints, or increased tolerance to genomic instability may be selected by radiation exposure [153,154,155]. Although not yet completely understood, radiation may induce immunologic reprogramming of the tumor microenvironment that can both promote radiosensitivity and resistance. Rather than continued anti-tumoral immunity following treatment, tumor growth can be promoted or the anti-tumor response modulated through upregulation of certain immune checkpoint ligands, recruitment of suppressive myeloid and tumor-associated macrophage populations, increased regulatory T cells, and induction of type 1 interferon signaling [83]. The role of the immune system highlights the need for immune-competent models and a better understanding of the dose-dependent molecular mechanisms during pre-clinical TAT studies. Clinically, this adaptive radiation resistance may cause reduced response and efficacy to repeat TAT administrations, and unopposed immune adaptation may limit sustained disease control.

On top of the physical and biological constraints, clinical trial feasibility and scalability remain significant challenges, largely due to limited patient recruitment with the relevant clinical trials restricting eligibility to minimal residual disease following cytoreductive surgery. Many have been single-center trials that limit patient generalizability, but multi-center trials are hindered by regulatory barriers, isotope availability, and the need for specialized infrastructures and personnel [156,157]. Furthermore, the global supply of alpha-emitters remains severely constrained by the production and purification processes that rely on specialized production facilities and complex regulatory frameworks [158]. Many of the isotopes investigated for TAT in OC listed in this review face significant production limitations. For example, ^211^At requires access to high-energy cyclotrons, and Bi-213 depends on the already constrained production of ^225^Ac [159,160]. Moreover, the current production of ^225^Ac and ^212^Pb is primarily concentrated in the US and Europe, resulting in high manufacturing costs, logistical complexity, and geographical disparities. These clinical trial recruitment and supply chain limitations raise concerns around equitable access, standardized hospital safety protocols, reimbursement codes, the scalability of clinical trials, and long-term sustainability of TAT within standard gyno-oncology practice.

## 7. Future Perspectives and Opportunities

Future progress in TAT for metastatic OC depends on advancements across multiple domains, including targeting ligands, delivery systems, imaging and dosimetry, regulatory pathways, and emerging computational tools. Many of these developments are in direct response to the challenges identified in the previous section, focused on heterogeneous antigen expression, unpredictable distribution, dosimetry challenges, systemic toxicity, isotope availability, and clinical trial constraints. One of the major opportunities to overcome inter- and intra-patient antigen expression heterogeneity restricting consistent tumor uptake lies in next-generation targeting ligands. The small size and rapid penetration of Alfa-tagged nanoparticles and single-domain antibodies have the potential to reduce the cold regions within the hypoxic niches and stromal-dense regions of larger tumors that are currently unreachable with current TAT strategies [161,162,163,164]. Peptide ligands also offer rapid tumor penetration, low immunogenicity, low toxicity, and wide dispersion throughout the peritoneal cavity that may mitigate difficulties associated with post-operative adhesion and turbulent peritoneal fluid flow patterns [165,166]. Given that solid tumors have established vasculature through angiogenesis, the novel hAnnA1 antibody that targets tumor vasculature in various cancers (i.e., ovarian, breast, prostate, lung, and liver) has promising potential to provide deeper and more uniform penetration [167,168]. Bi- and multi-specific antibodies that can engage two or more targets could increase the probability of improving the binding in tumors with downregulated or heterogeneous expression and may provide additional therapeutic mechanisms [169,170,171,172]. Key emerging bi-specific antibodies for recurrent OC are REGN4018 (Ubamatamab)^®^, undergoing phase 1/2 clinical trials, and LBL-033^®^, which has cleared FDA investigational pathways for clinical evaluation with the first human dosing performed in 2023 [173,174]. Both target MUC16 on cancer cells and CD3 on T cells that stimulate T cell-mediated killing. Combining the radiation sensitization property of T cells with TAT could further improve the treatment efficacy of OC patients. Moreover, ligands capable of carrying multiple chelators with rapid kinetics and high in vivo stability may allow for even higher energy deposition to compensate for tumors with low antigen expression to further improve therapeutic index [175,176,177]. With the exponential advances in protein engineering over the past decade, improvements in the targeting molecules and ligands have the potential to rapidly progress TAT for metastatic OC.

Optimizing how TATs are delivered addresses distribution and clearance challenges in IP administration. To achieve a more uniform distribution within the peritoneal cavity, biodegradable polymeric nanoparticles [178,179,180] and albumin-based carriers [181,182] are being explored for their ability to prolong residence time and reduce lymphatic clearance to improve delivery. Developments in these drug delivery vehicles may also buffer the recoil daughter radionuclides that may cause severe radiotoxic effects in organs (i.e., kidney) or lead to secondary tumorigenesis [183]. In addition to the containment of recoil daughters within the hydrophobic bilayer, liposomal encapsulation may also enhance cellular internalization by mimicking the body’s own cellular membrane to further improve dsDNA damage [184,185,186]. Nanoparticles (i.e., iron oxide composition) are being combined with magnetically steerable systems to more accurately guide directed delivery to the site of the tumors, especially as injected particles tend to accumulate at the bottom of the peritoneal cavity for immobile patients [187,188]. Sustained therapeutic exposure is being explored with an injectable hydrogel that forms deposits in situ and could gradually release alpha-emitters to minimize systemic activity peaks [189,190]. This is particularly appealing in post-operative settings where adhesion fragments the peritoneal space, causing a segmented distribution with IP delivery. Improvements in IP catheters and administration techniques that focus on better flow, less migration, and fewer complications may also be considered to optimize the spatial distribution, reduce complications, and improve the patient experience [191,192]. Addressing the non-uniform IP dose distribution of TAT would improve therapeutic efficacy, reduce toxicity, and simplify dosimetry modeling/calculations.

Exploring combination therapies with TAT could overcome tumor and microenvironment resistance factors that limit the effectiveness of monotherapy. Alpha-radiation that produces high-density dsDNA breaks could have synergistic potential with PARP or cell cycle checkpoint inhibitors as sensitizing agents, decreasing the effectiveness of DNA repair pathways and leading to even more dsDNA damage [193,194,195]. Cell lethality can occur at lower radionuclide doses with fewer adverse effects. Low-dose chemotherapy (i.e., platinum agents used in HIPEC) can also sensitize cells prior to TAT, but caution is needed with this strategy, as some agents may inadvertently increase drug resistance by promoting survival pathways or selecting for more resilient cancer stem cells [196,197]. TAT may create a favorable setting for immunotherapy (i.e., PD-1/PD-L1 and CTLA-4 blockade) by promoting immunogenic cell death, antigen presentation, and modulating macrophage and T cell populations within the peritoneal environment [198,199,200,201]. Alpha-radiation combined with CAR-T or CAR-NK therapies for solid tumors is currently under early investigation, where radiation has been shown to clear stromal barriers for better access to the tumor site, reduce immunosuppressive regulatory T cells, and potentiate effector cell infiltration for an abscopal effect [202,203]. Recently, combining targeting radionuclide therapy (^177^Lu-DOTA-trastuzumab) with gadolinium-based nanoparticles was shown to increase therapeutic effect and reduce the amount of injected activity needed in three different OC cell lines (SKOV3, OVCAR3, and A431) [204]. Combining strategies with TAT may be essential for overcoming the constraints of peritoneal metastases, even with antigen targeting and optimized delivery.

Innovations in imaging and dosimetry can be expected to address the unreliability of current organ-level dosimetry for alpha-emitters by transforming the clinical planning and safety estimations of TAT. Theranostic isotope pairs (i.e., ^203^Pb/^212^Pb, ^86^Y/^90^Y, ^111^In/^177^Lu) enable real-time quantitative SPECT or PET imaging of pharmacokinetics, allowing for dose planning based on individualized biodistributions rather than population estimates [205,206,207,208]. Furthermore, multi-chelator ligands that can carry both imaging and therapeutic isotopes allow for dynamic monitoring throughout the course of the treatment [209]. Personalized IP delivery and catheter placement strategies may be possible with advancements in peritoneal flow imaging using SPECT/PET mapping and fluorescence co-registration [210]. Other essential tools for predicting the toxicity and efficacy of TAT are next-generation micro-dosimetry technology (i.e., Voxel level Monte Carlo models and artificial intelligence-based reconstruction) for streamlined, accurate, and reproducible dosimetry [211,212,213,214].

Lastly, a precedent now exists for accelerating the development of TAT, as several radiopharmaceuticals have recently received FDA fast-track or breakthrough therapy designation (e.g., ^212^Pb-DOTAMTATE AlphaMedexTM, ^177^Lu-PSMA-617 Pluvicto^®^). A strong justification for expedited review would be a particularly relevant pathway for OC, given the unmet clinical need and limited treatment options. Coordinated multi-center phase 2/3 trials would be needed, but this would be restricted by the availability of alpha-emitting isotopes, stringent handling regulations, and the need for specialized facilities, as mentioned in the previous section. Standardized protocols (e.g., isotope handling, expansion of global generator supply chain/infrastructure, reimbursement frameworks) and collaborative networks (i.e., academic–industry partnerships, international consortia) are essential to broaden access and reduce the geographic limitation of patient enrollment in clinical trials [215,216]. Several technological approaches are being explored to address supply limitations, including accelerator-based production methods and a radionuclide generator system, offering opportunities to expand access. Public–private partner initiatives, such as programs supported by the U.S. Department of Energy and the International Atomic Energy Agency, are essential to expand isotope production capacity [217]. These regulatory and infrastructure advancements will collectively determine whether TAT can move from early-phase studies into widespread clinical practice for the treatment of OC and other peritoneal malignancies.

## 8. Conclusions

Looking forward, the platform developed through TAT for OC will naturally extend to other solid tumor malignancies (and vice versa) where microscopic peritoneal implants remain major drivers of mortality, including that of endometrial, cervical, gastric, pancreatic, and colorectal cancers [218,219]. The peritoneal cavity offers a unique anatomical and biological advantage for alpha-emitters with the confined environment that allows for localized high-LET radiation directly to microscopic implants while minimizing off-target toxicity. TAT is positioned to evolve into a broadly applicable modality for patients with advanced-stage peritoneal malignancies who currently have limited therapeutic options, with continued advancements in ligand engineering producing delivery vehicles to cover a variety of targets, reliable imaging/dosimetry, expanded generator production pipelines to stabilize global isotope supply, and efficient regulatory pathways. Integration of TAT into clinical practice as an adjuvant therapy or primary strategy for minimal residual disease has the potential to reshape the standard of care in the management of gynecological cancers and transform a historically deadly diagnosis into a therapeutically addressable target.

## Figures and Tables

**Table 1:** Alpha-emitter properties used in OC TAT. EC = electron capture. * In situ ^212^Bi generator.

Alpha-Emitter	Half-Life	Range (μm)	Emissions per Decay	Max Energy (MeV)
^224^Ra [28]	3.6 d	50–80	4 alpha	5.79
^225^Ac [29]	9.92 d	50–90	4 alpha, 2 beta-	5.83
^212^Pb * [30]	10.64 h	600	1 alpha, 2 beta-	8.375
^211^At [31]	7.21 h	55–80	1 alpha, 1 EC	5.982
^212^Bi [30]	60.6 min	40–100	1 alpha, 1 beta-	6.090
^213^Bi [29]	45.6 min	40–100	1 alpha, 2 beta-	5.869

**Table 3:** Summary of TAT OC clinical trials.

Agent (Target)	Alpha-Emitter	Route	Phase /Status	ID	Patient Population	Dosing Schema	Reported Toxicity/Primary Outcomes
MX35 F(ab’)_2_ (NaPi2b/SLC34A2)	^211^At	I.P.	Phase I completed, long-term follow-up	NCT04461457	Ovarian cancer patients in clinical remission, with microscopic residual	Escalating doses in the 20–215 MBq/L range	Toxicity was limited to transient, low-grade procedural symptoms; hematologic, renal, and late radiation-induced toxicities were not observed
Trastuzumab (HER2)	^212^Pb	I.P.	Phase I completed	NCT01384253	Patients with HER2+ ovarian and other peritoneal carcinomatosis	Escalating doses up to 27 MBq/m^2^	Minimal toxicity across the initial dose cohorts and no late toxicity observed in most patients with >1 year follow-up
Radspherin CaCO_3_ microparticles	^224^Ra	I.P.	Phase 1/2a completed; phase 2 randomized ongoing	NCT03732768;NCT06504147	Ovarian cancer patients undergoing cytoreductive surgery ± HIPEC with residual microscopic peritoneal disease	Escalating doses at 1–2-4–7 MBq not reported for ongoing trials	No dose-limiting toxicities across the 1–2–4–7 MBq dose levels, with one serious adverse event related to administration. The ongoing trial is intended to track safety/toxicity (adverse events) alongside efficacy

## Data Availability

Not applicable. No review protocol was prepared.

## Supplementary Materials

The following supporting information can be downloaded at: https://www.mdpi.com/article/10.3390/molecules31061019/s1, Supplemental File: Pre-clinical Evidence of TAT in OC.

## Abbreviations

The following abbreviations are used in this manuscript:

OC	Ovarian cancer
TAT	Targeted alpha-particle therapy
dsDNA	Double-stranded DNA
RIT	Radioimmunotherapy
HIPEC	Hyperthermic intraperitoneal chemotherapy
LET	Linear energy transfer
FIGO	International Federation of Gynecology and Obstetrics
IP	Intraperitoneal
IV	Intravenous
TFF	Tumor-free fraction
IC	Initial activity concentration
mCRPC	Metastatic castration-resistant prostate cancer
