# Supplementary material for: A Scoping Review of the Challenges and Future Perspectives in the Use of Alpha-Emitters for Metastatic Ovarian Cancer
Source: Molecules. 2026 Mar 18;31(6):1019. doi: 10.3390/molecules31061019 (PMC13029560; doi:10.3390/molecules31061019)
Supplement: Supplementary file 1 [file molecules-31-01019-s001.zip › molecules-4155550-supplementary.pdf]

Supplemental Pre-clinical Evidence of TAT in OC

S1. ^{211}At labelled Anti-FR-α

One of the earliest proof-of-concept study with TAT for metastatic OC was using ^{211}At ($t_{1/2}$=7.2 hours) labelled monoclonal antibodies directed against folate receptor-α (FR-α), one of the most consistently overexpressed antigens in epithelial OC. Three major studies evaluating ^{211}At-labelled humanized (farletuzumab) and murine (Mov18) FR-α antibodies have demonstrated promising therapeutic potential when IP delivered against microscopic peritoneal OC. In the first study, nude mice were IP (intraperitoneal) inoculated with OVCAR3 cells and half of them were IP injected with 450-555 kBq of ^{211}At-Mov18 14 days later [94]. The treated group showed significant long-term survival benefits, achieving a median survival of 213 days vs. 138 days in controls, with 33% exhibiting complete histologically confirmed cures. Another treatment was performed for the advanced stage of the model with ascites, and 10 of these mice were IP injected with 377-389 kBq of ^{211}At-MOv18. Results showed delayed ascites accumulation, suggesting a palliative benefit. In a later farletuzumab study, BALB/c nude mice were IP implanted with OVCAR3 cells to generate disseminated microtumors prior to receiving 170 kBq/mL of ^{211}At-farletuzumab via IP infusion at activity concentrations scaled to human dosimetry [95]. This therapy produced 91% TFF compared to 9-14% in control groups of PBS, unlabeled farletuzumab, and unspecific ^{211}At-rituximab with a 6-10 folds increase in anti-tumor efficacy. Additionally, alpha-camera imaging confirmed high homogeneous uptake across tumors with ~300 μm in size, expected to be sterilized with an assumed eradicative dose of 10 Gy. This same group performed a follow-up study comparing 300-400 kBq of IP or IV administered ^{211}At-MOv18 with similarly promising results using nude mice implanted with OVCAR3 cells modelling post-debulking peritoneal micro-metastases [96]. At 6 weeks post-treatment, TFF were 90% of the IP ^{211}At-MOv18, 40% for IV ^{211}At-MOv18, and 0-25% for the remaining IP unlabeled Mov18, ^{211}At-C242, and PBS controls. No obvious side effects or a significant depression of white blood cells was observed. All studies described above demonstrate that IP delivery of ^{211}At labelled FR-α antibodies can selectively target microscopic OC implants in mouse models that closely mirror human OC disease distribution in the peritoneal cavity, deliver curative doses, and improve survival. Toxicity was minimal in both studies and activity levels within the projected tolerance for clinical translation. Limitations include the limited antibody

penetration in nodules exceeding a few hundred microns, the difference in peritoneal clearance between mice and humans during translation, and FR-α expression heterogeneity that could affect treatment efficacy, all of which did not diminish the overall translational promise of TAT. These initial studies established several principles to guide later TAT development: microscopic tumor size impacts homogenous uptake, IP administration maximizes tumor exposure while limited systemic toxicity, and clinically scalable activity levels can achieve robust tumor control.

S2. ^{213}Bi labelled Anti-MUC1 and Anti-CD138

Building upon the success of ^{211}At, investigators explored the shorter half-life alpha emitter, ^{213}Bi ($t_{1/2}$=45 mins), targeting MUC1 (C595 monoclonal antibody) and CD138 (B-B5 monoclonal antibody). In the ^{213}Bi-C595 study, MUC1 was strongly expressed in 73% of 26 OC tissues from both primary and metastatic sites [97]. OVCAR3 ascites models using BALB/c nude mice were IP administered 355, 710, or 1065 MBq/kg 9 days post cell inoculation. A single dose as low as 355 MBq/kg prolonged survival for up to 25 days compared with controls. Pharmacokinetic analyses demonstrated a nearly six-fold higher activity in tumors compared to blood (tumor: blood ratio of 5.8) in biodistribution studies, and a maximum tolerated dose >1180 MBq/kg at 21 weeks with only mild radiation nephropathy observed. In the complementary study evaluating ^{213}Bi-B-B4, a SHIN-3 post-operative peritoneal carcinoma model was used to mimic residual microscopic dis-ease post-cytoreductive surgery [98]. 7.4 or 11.1 MBq of ^{213}Bi-B-B4, cisplatin only, and combination therapy were IP injected to nu/nu mice 3 days after SHIN-3 engraftment. Results showed that 7.4 and 11.1 MBq produced significantly improved survival (63.6-71.4% alive at 90 days vs. 10% in untreated controls) along with marked reductions in the maximal average bioluminescence (0.45×10^7 vs. 2.7×10^7 counts) and peritoneal carcinomatosis index. Toxicity studies showed insignificant reduction in platelets and white blood cells for all treatment groups and mice treated with ^{213}Bi-B-B4 recovered their initial weight. Limitations for these studies include small cohort sizes, the use of immunodeficient murine models that may not accurately capture the tumor microenvironment, heterogeneity of antigen expression amongst OC cell lines, and logistical challenges of working with a very short half-life radioisotope. Nonetheless, both studies support ^{213}Bi-based TAT as a robust approach for eradicating microscopic

peritoneal metastases from MUC1 and CD138 expressing OC and controlling associated malignant ascites.

S3. ^{212}Pb labelled Anti-B7-H3

Investigators began targeting B7-H3 antigen associated with aggressive tumor behavior and cancer-initiating cells (CIC) to address OC resistance and recurrence. One study has also explored the direct targeting of B7-H3 antigen in athymic nude mice IP implanted with ES-2 or A2780cp20 cancer cells treated with 376.96 monoclonal antibody radiolabeled with ^{212}Pb ($t_{1/2}$=10.6 hours) [99]. This study had a separate cohort that modeled chemotherapy-resistant environments and recurrent disease using CIC-enriched tumors. A single IP injection of ^{212}Pb-376.96 was administered at doses ranging from 0.17-0.7 MBq at day 4 for the ES-2 model and day10 for A2780cp20 model. Biodistribution showed preferential uptake in ascites and tumors with low off-target uptake suggesting a favorable therapeutic index. The treatment results collective showed that ^{212}Pb-376.96 inhibited clonogenic survival of both cell lines and CICs up to 40 times more effectively than isotype-matched controls, with corresponding improvements in median survival at 2-3 times higher than untreated or non-targeted groups. Toxicity studies were limited despite the promising treatment efficacy, so future studies would require more comprehensive hematologic and organ-level evaluation prior to clinical translation. These findings were particularly significant because they suggested that B7-H3 directed TAT could overcome one of the central challenges to OC remission and supports the further development as an adjuvant strategy following standard interventions.

S4. ^{225}Ac and ^{213}Bi labelled Anti-HER2

One of the most extensively explored targets for TAT in OC has been against the HER2 receptor with a combination of monoclonal, pre-targeting bi-specific, and single domain fragment antibodies labeled with a variety of α-radioisotopes (e.g., ^{225}Ac and ^{213}Bi). HER2 has been found to be a promising therapeutic target as this receptor is overexpressed across aggressive epithelial OC. *In vitro* binding results with these HER2-targeting vectors have demonstrated selective, high-affinity binding. Early work using labeled single-domain antibody fragments (HER2-targeting ^{213}Bi-DTPA-2Rs15d and non-

targeting control ^{213}Bi-DTPA-R3B23) demonstrated rapid tumor uptake 15 mins following IV injection with low uptake in normal tissue [100]. Potent cytotoxicity against peritoneal SKOV3 xenograph models was observed along with significant suppression of ascites formation and increased survival. Three cumulative IV injections of both 0.5 and 1 MBq of ^{213}Bi-DTPA-2Rs15d resulted in significantly longer median survival of 80 and 67 days, respectively, compared with 53 days for the 0.9% NaCl control group. The only notable toxicity was minor renal tubular degeneration and dilation. Some of the most compelling pre-clinical OC TAT outcomes have been observed in another study evaluating a pre-targeting approach with a HER2-targeting bispecific antibody followed by ^{225}Ac-Proteus-DOTA ($t_{1/2}$=9.9 days) [101]. Nude mice with diffuse SKOV3 peritoneal metastases first received an IP injection of the unlabeled bispecific antibody and IV injection of a clearing agent prior to receiving either 1 or 2 cycles of 37 kBq of ^{225}Ac-Proteus-DOTA treatment. Treatment results demonstrated significantly prolonged median survival (154 vs 112 days) and lower bioluminescence signal (0.97 vs 18737 AUC), compared with controls. Mild tubular changes without functional impairment were observed in the 2-cycle treatment group. Key limitations include variable vector internalization due to different levels of HER2 expression on cell lines and low-grade renal toxicity that require careful consideration. Nonetheless, these two pre-clinical studies collectively demonstrate that HER2-targeting TAT are effective at reducing the tumor burden and eliminating microscopic peritoneal metastases in OC and have progressed to clinical trials as described in the following section.

S5. ^{211}At labelled Anti-NaPi2b

NaPi2b (SLC34A2) TAT with MX35 has been another extensively explored target for OC evaluated in a series of OVCAR3 xenograft models using a variety dosing paradigms, including single-dose, fractionated, and pre-targeted. Initial studies with single dosing have consistently achieved high TFF using a micro-metastatic intraperitoneal OC disease model with OVCAR3 implanted nude mice. In a dose escalation study using ^{211}At-MX35 F(ab′)2, significant increase in treatment efficacy was observed in IP treated groups with 25%, 22%, 50%, and 61% for 25, 50, 100, or 200 kBq compared to 0% TFF in the unlabeled antibody controls [102]. Microscopic tumors, macroscopic tumors, and ascites were also reduced to 30-40% in the treated groups from 100% in controls. This same group performed another dose escalation study using IP administered 400, 800, or 1200 kBq

^{211}At-MX35 compared with unlabeled MX35, PBS, and 400 kBq ^{211}At-Mov18 controls [103]. Results showed high efficacy with ^{211}At-MX35 where 3/25 mice developed ascites and 8 had microscopic growth. This was comparable to the ^{211}At-Mov18 positive control, where no mice developed ascites and only 3/10 having microscopic tumors. The groups with unlabeled MC35 or PBS had all mice develop ascites and microscopic tumors, along with 6/9 with macroscopic tumors. Another related study using 400 kBq of ^{211}At-MX35 F(ab′)2 or non-specific ^{211}At-Rituximab demonstrated the relevance of the tumor size and penetrating ability of antibodies and or their fragments [104]. For larger tumors where the radius was greater than the maximum path length of ^{211}At, specific targeting and the smaller size of MX35 F(ab′)2 provided a higher mean absorbed dose >22 Gy compared with non-specific Rituximab <6Gy. Using the same MX35 antibody, a separate group found that labeling with ^{211}At was more effective than ^{213}Bi at decreasing the micro-metastatic burden of OC [105]. TFF were 60% and 90% for ~2.7 MBq of ^{213}Bi-MX35 and ~0.44 MBq ^{211}At-MX35, respectively. Building on these findings, two fractionated dosing studies were initiated with mixed findings. In the first fractionated study, repeated treatments of 400 kBq ^{211}At-MX35 F(ab′)2 starting from 3 repeats every 7th day significantly increased the therapeutic efficacy without observed toxicity [106]. TFF increased from 17% to 39% from single to 3 fractions and eliminated ascites for groups given >5 fractions. The other study found no therapeutic advantage of fractionated dosing when the total activity of the fractions was matched with the single dosing [107]. TFF were 56% and 39% when treated with 800 kBq and 400 kBq, respectively. TFF of fractionated dosing with 3×267 kBq and 3×133 kBq was lower at 41% and 28%, respectively. An advantage to fractionation was the reduced myelosuppression. Pre-targeted TAT is the third strategy that have been explored using avidin-MX35 and ^{211}At-B-PLsuc [108]. Although the TFFs are both 45%, 1.5 MBq of the pre-targeted avidin-MX35 and ^{211}At-B-PLsuc had a lower incidence of ascites and macroscopic tumors >1mm compared with conventional TAT at 0.9 MBq. Key limitations across these studies include testing only the OVCAR3 cell line given and using immunodeficient models that could under-estimate the effectiveness of fractionated therapy [109]. Together, these studies showed that single dosing of ^{211}At-MX35 targeting NaPi2b is effective at eliminating microscopic peritoneal OC, particularly when the tumor size is within range of energy emission or optimized with pre-targeting. These promising results have led to subsequent clinical trials as outlined in the next section.

S6. ^{225}Ac labelled Anti-TAG-72

Another target that has been explored in TAT for OC is the tumor-associated glycoprotein 72 (TAG-72) that is found on about 90% of epithelial OC cells [110]. A pre-clinical evaluation of TAG-72-directed ^{225}Ac-labeled-DOTAylated-huCC49 ($t_{1/2}$=9.9 days) was performed in the OVCAR3 xenograph model using female NOD/SCID mice with subcutaneous tumors [111]. Prior to treatment, ^{64}Cu-CC49 PET imaging and biodistribution were performed, which confirmed high tumor targeting of up to 26% ID/g at 46 hours post-injection. The radiolabeled huCC49 also retained high immunoreactivity >85% with labeling efficiency >89%. Escalating intravenous (IV) doses of ^{225}Ac-huCC49 (1.85, 3.7, or 7.4 kBq) or fractionated treatment regimen (1.85kBq initial dose with five weekly 0.7 kBq doses) were administered. Results showed that the single-dose therapy produced a dose-dependent tumor growth suppression and improved survival, where the 7.4 kBq dose tripled mean survival compared with the untreated controls (103 vs 32 days). The fractionated strategy that aimed for consistent intratumoral activity achieved comparable therapeutic efficacy, with survival extended up to 86 days. In general, negligible systemic toxicity was observed in both treatment strategies; however, the highest single dosing showed some early signs of toxicity. This study provides compelling evidence that TAG-72-directed ^{225}Ac TAT, particularly with a fractionated treatment regimen, has a prolonged and potent antitumor effect for the treatment of OC and other solid tumors. Prior to the eventual clinical transition, further evaluation with IP-immune competent OC models is required to accurately represent the disease.

S7. ^{225}Ac labelled Anti-MUC16/CA126

Most recently, pre-clinical TAT OC studies targeting fully glycosylated and hypo-glycosylated isoforms of the oncoprotein MUC16 (also known as CA126) have been explored using both alpha- and beta-particle platforms based on a humanized monoclonal antibody, huAR9.6. A TAT study evaluated the direct (0.037 MBq ^{225}Ac-mcp-PEG8-AR9.6) and a pre-targeted click ligation (AR9.6-TCO followed by 0.148 MBq ^{225}Ac-mcp-PEG8-Tz 72 hours later) of ^{225}Ac labelled huAR9.6 through IV administration in subcutaneous OVCAR3 xenograph models in nude mice [112]. Although there was no difference in response between the delivery platforms, both ^{225}Ac-labelled huAR9.6 treatments produced robust anti-tumor responses with no tumor recurrence throughout

the study, and overall survival was significantly higher than in the saline and IgG control groups. Direct administration was found to be more effective on a cellular level, with more frequent γ-H2AX signal during immunostaining. Renal tubulo-nephropathy, ovarian atrophy, and transient hematological toxicity were observed. In contrast, an earlier beta-emitter study by the same group used ^{177}Lu-CHX-A99-DTPA-huAr9.6 in subcutaneous ovarian cancer xenograph modes (OVCAR3 and OCVAR5) in immunodeficient mice with single IP doses at 9.25, 18.5, and 27.75 MBq [113]. Potent anti-tumor effects were seen in all cohorts compared to control groups (saline and ^{177}Lu labelled IgG), with significantly higher overall survival in the 9.25 18.5 MBq cohorts. 9 out of the 15 tumors in the lowest dose demonstrated recurrence at 5-10 weeks post-treatment. Dose-dependent hematologic toxicity was observed with higher activities associated with weight loss and cytopenias. Key limitations across both studies include the reliance on subcutaneous models that cannot accurately capture peritoneal metastases and the dose-limiting off-target toxicity. While ^{177}Lu-based beta-emitter treatment provides target-dependent tumor control, ^{225}Ac-based TAT offers superior and durable eradication of MUC16-expressing OC at lower doses.

S8. ^{212}Pb labelled Anti-PTK7

Another recent OC target for TAT is a receptor found in tumor-initiating cells, protein tyrosine kinase 7 (PTK7), using IP administered OI-1 antibody labelled with ^{212}Pb. In the proof-of-concept study, a chimeric human IgG1 version (chOI-1) antibody was developed to test the therapeutic efficacy in disseminated IP SKOV3 xenograft models [114]. On day 3 post-inoculation, a single IP bolus of 0.18 or 0.405 MBq of ^{212}Pb-TCMC-chOI-1 was administered compared to saline, chIO-1, and 0.211 or 0.384 MBq ^{212}Pb-TCMC-hIgG controls. Both targeted dose levels produced significant reduction in tumor burden and increase in TFF of 100% (0.18 MBq) and 87.5% (0.405 MBq) vs 25% for the non-targeted radiolabeled control. Toxicity was not investigated in this study. A second OC peritoneal model (A2780) was evaluated by another group and implemented on-site ^{212}Pb production with a two-step ^{224}Ra generator [115]. IP administration of treatments was performed 18 days post-inoculation with 0.458 MBq of ^{212}Pb-TCMC-chOI-1 compared to 0.47 MBq of free ^{212}Pb, 10 μg of unlabeled chOI-1, or a vehicle buffer control. ^{212}Pb-TCMC-chOI-1 (42 days) significantly improved survival compared with vehicle control (25.5 days). The treatment appeared to be well tolerated based on body weight assessment, but

no toxicology assessments were performed for definitive conclusion of safety. Although toxicity results are limited, both studies support PTK7-directed TAT to reduce the peritoneal micro-metastatic burden from OC. These pre-clinical studies from the past two decades collectively describe a coherent story that TAT is uniquely suited for the biology and spatial distribution of OC metastases within the peritoneal cavity. The consistency of these findings provides a compelling reason for continued clinical translation that are underway, as highlighted in the following section.
